# Association of Physiological Performance, Physical Fitness, and Academic Achievement in Secondary School Students

**DOI:** 10.3390/children11040396

**Published:** 2024-03-27

**Authors:** Umut Canli, Monira I. Aldhahi, Hamza Küçük

**Affiliations:** 1 Sports Science Faculty, Tekirdag Namik Kemal University, Suleymanpasa, Tekirdag 59010, Turkey; ucanli@nku.edu.tr; 2 Department of Rehabilitation Sciences, College of Health and Rehabilitation Sciences, Princess Nourah bint Abdulrahman University, P.O. Box 84428, Riyadh 11671, Saudi Arabia; 3 Yasar Dogu Faculty of Sport Sciences, Ondokuz Mayıs University, Samsun 55270, Turkey; hamza.kucuk@omu.edu.tr

**Keywords:** blood pressure, heart rate, academic performance, vertical jump

## Abstract

This study aimed to compare the physiological performance and physical fitness based on the academic achievement levels of secondary school students and to explore the effect of gender on the relationship between physiological performance, physical fitness, and academic achievement. In this cross-sectional study, 304 children aged 13–14 years were recruited. To assess physical fitness, students performed a 20 m sprint test, a pro-agility test, a one-mile endurance run/walk test, and a countermovement jump test. At the end of the one-mile endurance run/walk test, the estimated VO_2peak_ value of the participants was calculated. The physiological performance of the students was determined by measuring their resting heart rate and blood pressure. Students were grouped into three categories based on their academic achievement levels. The assessment of academic achievement considered their scores from the previous academic year. The scores were divided into three levels: poor (average score of 69 points or less), average (scores ranging from 70 to 84 points), and good (scores of 85 points or higher). The study revealed a notable disparity among students’ VO_2Max_ measurements based on their academic achievement (F = 8.938, *p* < 0.001, η^2^ = 0.056). However, we observed that the group with poor academic achievement displayed lower diastolic blood pressure values than the groups with average and good performances. Finally, no significant gender differences were evident in the relationship between academic achievement and any of the physical and physiological parameters.

## 1. Introduction

The evaluation of adolescent students’ overall school success often revolves around their academic achievement, which is typically measured through exam scores [1]. It is known that parents and all other dynamics in the education system acknowledge such an assessment approach. However, it is crucial to acknowledge that multiple variables can influence this approach [2]. Previous research has identified several factors, such as learning pace, intelligence, self-esteem, personality traits, self-efficacy, motivation, study habits, parental attitudes, socio-economic status, and school type, that contribute to these academic outcomes [3,4,5,6].

Although there is growing interest in comprehending the variables that affect academic achievement, the ongoing drop in achievement continues to be of great concern to the academic community [7]. In this sense, more evidence is needed regarding the different variables associated with academic achievement.

Physical fitness is recognized as another important element affecting academic achievement or achievement-related variables, in addition to the previously stated variables. Numerous studies have investigated the relationship between physical exercise and academic achievement, cognitive processes, and physical fitness in children and adolescents [8,9,10,11]. Various mechanisms have been proposed to explain the impact of physical activity on the brain structure and function. Additionally, recent evidence highlights that regular exercise or physical activity can lead to improved learning ability, physical fitness, and, subsequently, enhanced brain function and academic performance [12,13,14]. Furthermore, physical activity has been shown to optimize cell capillaries, increase blood flow, enhance brain oxygenation and neurotransmitter and neurotrophic levels, promote nerve cells, and increase brain tissue volume, which in turn leads to new nerve cell connections [15,16,17]. Previous findings suggest that these physiological changes in the brain positively affect concentration, memory, and processing strategies [18] and that such impacts are more likely to contribute to overall school success, as executive functions and cognitive control promote learning [19]. It was reported that physically fit children would have better executive functions and engage in healthier behaviors, ultimately leading to school success [20].

Studies have examined physical fitness and academic achievement in children and young students [21,22,23,24,25,26,27,28,29]. Various investigations involving sample sizes ranging from 134 to 884,715 participants aged 7 to 15 years have been conducted [25,28]. These studies utilized the US Fitnessgram, a comprehensive test suite that includes cardiovascular endurance (PACER), muscular endurance (push-ups and sit-ups), flexibility (sit and reach), and body mass index assessments; the endurance was measured using US Fitnessgram and a battery of tests [25,28]. In a study by Eveland-Sayers et al. [26], the standard PACER test was substituted with a one-mile run. A battery test measures encompassed lung function, a 50 m sprint for muscular power, a 1.6 km run for cardiorespiratory endurance, a standing long jump for muscular power, and sit-ups and push-ups for muscular endurance [29]. One study utilized the evaluation of academic competence by a school official on a five-point scale, while three others employed standardized tests to measure academic performance. Two studies factored in parental education and socio-economic status in their analysis [25,29]. A consistent, moderately positive link was established between physical fitness and academic performance across the studies, with cardiovascular fitness exhibiting the strongest correlation, demonstrated by r values from 0.41 with the PACER test to 0.20 for a lengthier one-mile run. Additionally, correlations between academic achievement and measures of muscular force/power and flexibility have been observed [30]. The findings of our study are anticipated to enrich the existing body of literature in this field. There are only a few studies in the relevant literature that look at the effects of factors like blood pressure and heart rate on young people’s academic achievement. Students with high blood pressure outperform their counterparts in terms of intellectual achievement, according to Berendes et al. [31]. However, other studies disagree with these findings, suggesting that additional evidence is needed to substantiate this relationship [32,33]. Therefore, a comprehensive study exploring the relationship between academic achievement, blood pressure, heart rate, and physical fitness among young students would significantly contribute to the existing literature. Additionally, analyzing the impact of gender on these variables could enhance the scholarly value of this research. This study aims to compare the physical and physiological performance levels of secondary school students based on academic achievement and to examine the influence of gender on the association between physical and physiological performance and academic achievement.

## 2. Materials and Methods

### 2.1. Study Design and Participants

This study employed a cross-sectional design based on a non-experimental methodology. This research design is a type of research study in which a group of people is observed or certain information is collected at a single point in time or over a short period [34]. The sample consisted of 304 children aged 13–14 years who were enrolled in seven public schools in Tekirdag, Turkey. The schools where the participating groups were located were in the city center, and these schools had similar socio-economic family structures. These students were all studying in the eighth grade of secondary school. The sample size was calculated as at least 276 students, with a margin of error of 5%, a z-score equal to 1.96, and a confidence index level set of 95% [35]. A total of 304 participants participated in the study. We stratified the participants into three groups based on their academic achievement scores (the criteria proposed for classifying academic achievement are specified below). The study’s inclusion criteria were being 13 or 14 years old, in the eighth year of secondary school, and not having a cardiovascular, orthopedic, or neurological condition. The exclusion criteria, on the other hand, were specified as functional illiteracy, a history of attention deficit hyperactivity disorder, intellectual disabilities, visual or hearing impairment without correction, and an inability to participate in physical assessments. However, 23 students who did not meet the stated conditions for participation were excluded from the study. The participants and their parents were informed of the experimental procedures and provided written consent. This study was performed in accordance with the ethical standards of the Declaration of Helsinki and ethical approval granted by the Ethics Committee of Tekirdag Namik Kemal University (The ethical approval code: 2021.85.04.03; Date: 13 April 2021).

### 2.2. Procedure

**Figure 1:**
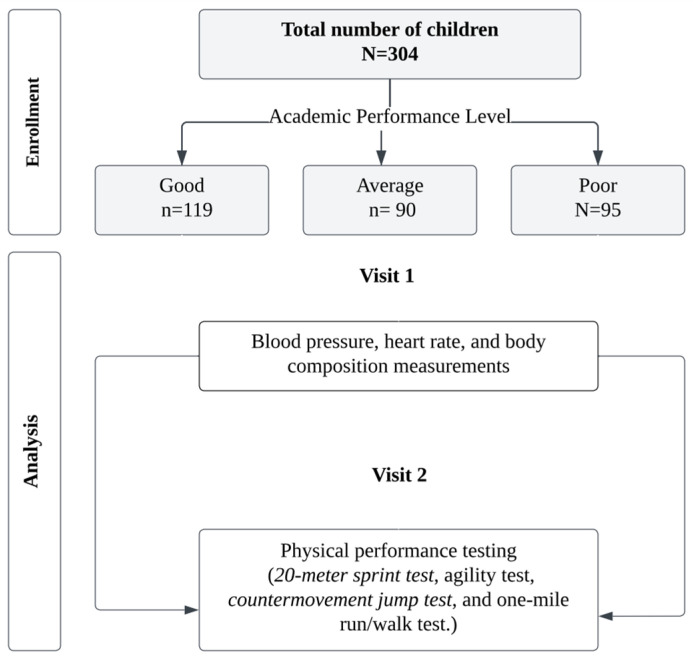
Schematic representation of the study flow diagram.

All measurements were performed in the same order by the same researchers. All tests were performed at the same time of the day (11:00 a.m.–14:00 p.m.) to avoid the influence of circadian rhythms on the study results. Participants attended two sessions: anthropometric measurements, physical fitness tests, and physiological performance tests. During the first session, the blood pressure, heart rate, and anthropometric measurements were obtained from the participants. In the second session, all physical fitness tests were conducted using the following speed tests: agility test, vertical jump test, and one-mile run/walk test. Prior to testing, the protocols were explained and demonstrated to the participants. Before the high-intensity activities and tests, a standard warm-up consisting of 5 min of jogging followed by 5 min of dynamic stretching was performed. After the tests were completed, the participants performed cooling exercises. A minimum 24 h break was taken between sessions. Figure 1 shows a schematic of the study flow diagram.

### 2.3. Instruments

#### 2.3.1. Body Composition Assessment

The body composition assessment included the measurement of height using a Mesilife 13539 portable stadiometer; Istanbul, Turkey) [36]. The participants’ body weights were quantified in kilograms to a precision of 0.01 kg utilizing a PoloSmart PSC05 Mood Glass Digital Scale [37]. The body mass index (BMI), expressed in kg/m^2^, was subsequently calculated by dividing the body weight by the square of the height.

#### 2.3.2. Physiological Performance Tests

Resting heart rate assessment: The participants’ resting heart rates were measured using a telemetric heart rate sensor (Polar Verity Sense; Kempele, Finland). After wearing the heart rate sensor, the participants remained in a sitting position for 20 min, and the lowest heart rate measured in the last 5 min was recorded as the resting heart rate (RHR). While the participants were waiting in a sitting position, they were not allowed to do any activity that could increase their heart rate (talking, playing with their mobile phone, etc.) [38].

Blood pressure measurement: Blood pressure measurements are critical tools for monitoring individuals’ cardiovascular health and detecting potential problems early. The blood pressure was assessed using an Omron HEM 742 automated oscillometric sphygmomanometer (Kyoto, Japan). The protocol was created by the American Heart Society [39] and was used to measure the blood pressure of the participants.

#### 2.3.3. Physical Fitness Tests

20 m sprint test: This is considered an essential performance metric critical to success in sports that involves sprinting due to its ability to measure an athlete’s speed and explosive power over short distances [40,41]. The 20 m distance was chosen for one reason: this distance was part of the subjects’ regular fitness-testing battery [42]. The best time was recorded using a photocell gate (Newtest Powertimer 300-series, Oy, Helsinki, Finland). The assessment was initiated by instructing the students to position their leading foot 0.2 m away from the initial photocell gate while standing. The test was performed twice with a rest interval of 2–3 min between each trial. Statistical analysis considered the shortest recorded time in seconds.

Pro-agility test: This is a popular agility test used to assess an individual’s ability to quickly change direction. The test involves sprinting back and forth between three designated points, measuring the time taken to complete the course. The setup included placing three cones in a straight line five yards apart. The cones are arranged in the following order: Cone A (starting point), Cone B (middle point), and Cone C (finishing point). Before starting the test, we ensured that the participants had an adequate warm-up to prevent injuries. The front foot was aligned with the cone. On the “go” command, the participant sprints as quickly as possible to Cone B, touching the ground or the cone with their hand before changing direction. After touching Cone B, the participant immediately changed direction and sprinted to Cone C, again touching the ground or cone with their hand. Once the participant reached Cone C, the timing was stopped, and their time was recorded [43]. Similarly, we recorded the test times using a photocell gate (Newtest Powertimer 300-series, Oy, Finland). The test was applied 2 times, and a 2 min rest interval was provided between the two tests. The test protocol was demonstrated by researchers in practice. The shortest recorded time represented the agility performance of the participants.

1-Mile Endurance Run/Walk Test: This test is commonly adopted for measuring aerobic endurance and aims to record the fastest possible time to complete a one-mile run. Participants were asked to run as fast as possible in a short time. The time taken to complete the mile was recorded in minutes and seconds [44]. The heart rate variability of the participants during the/run/walk test was monitored using an optical heart rate monitor (Polar Verity Sense, Kempele, Finland). The Bluetooth^®^, ANT+, internal memory, and Polar Verity Sense are connected to an app, and data are saved in the app.

The following equations were used to estimate the oxygen update (VO_2Max_):

Male (VO_2Max_) = 108.844 − 0.1636 W − 1.438 T − 0.1928 HR

Female (VO_2Max_) = 100.5 − 0.1636 W − 1.438 T − 0.1928 HR

W = weight in kg, T = time for the one-mile run, and HR = heart rate at the end of the run [45].

Countermovement jump test (Determination of Estimated Anaerobic Power): The countermovement jump (CMJ) is commonly used for the assessment of lower-body mechanical capacities. It has been used to monitor sports performance [46], inter-limb asymmetries [47], neuromuscular fatigue [48], and the effectiveness of different training programs [49]. The participants’ countermovement jump heights were measured using a Myotest Pro system accelerometer (Myotest SA, Sion, Switzerland). The device was attached to the belts and fixed vertically. Next, they were instructed to attempt to reach their maximum jump height when hearing the signal sound [50]. They were also instructed to maintain the knee joint at approximately 90° for each jump condition [51]. The highest jump score from the three trials was used for the evaluation. The test protocol was demonstrated by the researchers in practice. The vertical jump test was used to measure anaerobic power, with the participants performing the test protocol three times with 1–2 min rest between each trial [52].

P (kg-m/s) = √4.9 (body weight) × √vertical jump height

#### 2.3.4. Academic Achievement

To determine the participants’ academic achievement levels, we considered the average achievement scores from the previous academic year. Typically, this score is calculated by dividing the total final exam grade of all courses by the number of courses. We obtained the participants’ achievement scores from the administrative registry office of their schools. The scores were categorized into three levels of academic achievement: poor (average ≤ 69 points), average (70 points ≤ average > 84 points), and good (≥85 points) [53] (Figure 1). This methodology ensured a thorough and accurate assessment of participants’ academic achievement levels. Academic score averages representing students’ academic achievement levels were given by the school administration.

### 2.4. Statistical Analyses

Descriptive statistics were used to define the sample characteristics. Next, we considered skewness-kurtosis values to determine whether the research data were normally distributed. As a rule of thumb, Tabachnick and Fidell proposed that the data with skewness-kurtosis values between −1.5 and +1.5 show a normal distribution [54]. For normally distributed data, we analyzed the mean differences between groups using one-way analysis of variance (ANOVA) with the Bonferroni correction and calculated the eta-squared (η^2^) to reveal the effect size of any significant difference between the variables. Moreover, we calculated Pearson product-moment correlation coefficients to investigate the possible associations between the variables. Finally, we used the Fischer *r*-to-*z* transformation to analyze the significant differences between the correlation coefficients by gender. This transformation often seeks to convert correlation coefficients to standardized values (*z*-values) to make them comparable (the relevant formula is presented below) [55]. All statistical analyses were performed using SPSS version 18.0 (SPSS, Chicago, IL, USA), and the statistical significance was set at *p* < 0.05.

## 3. Results

**Figure 2:**
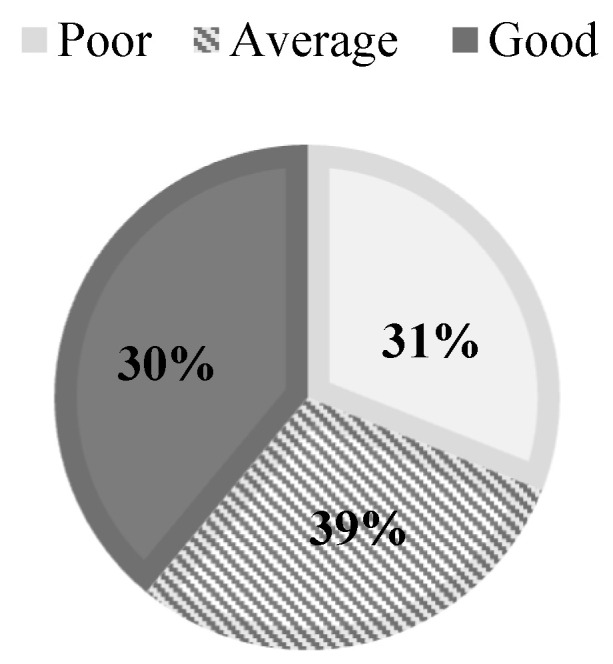
Sample distribution by academic performance.

The distribution of the samples according to age and gender is presented in Table 1. The data reveal that the largest proportion of students are at the age of 13. Figure 2 shows the percentage of students based on their academic performance levels.

Table 2 shows the descriptive statistics of the findings concerning the RHR, blood pressure, and physical fitness variables. Additionally, a comparison of the specified variables in terms of academic performance level is presented.

**Figure 3:**
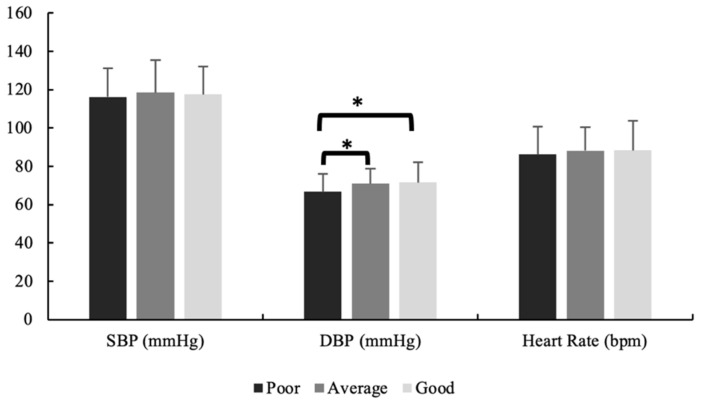
Systolic and diastolic blood pressure (SBP and DBP, respectively) and heart rate were measured by academic achievement (* denotes *p* < 0.05).

The findings showed no significant difference between the participants’ systolic pressure and heart rate values according to their academic achievement (F = 0.583, *p* > 0.05; F = 0.717, *p* > 0.05, respectively). However, we found a significant difference between the groups in their diastolic pressure values based on academic achievement (F = 7.696, *p* < 0.001, η^2^ = 0.049). We used the Bonferroni correction to determine the source of the significant difference within the groups, and it was found that the diastolic pressure value of the poor academic achievement group was markedly lower than that of the average academic achievement (66.7 vs. 70.9; *p* < 0.01) and the good academic performance groups (66.7 vs. 71.5; *p* < 0.001) (Figure 3).

**Figure 4:**
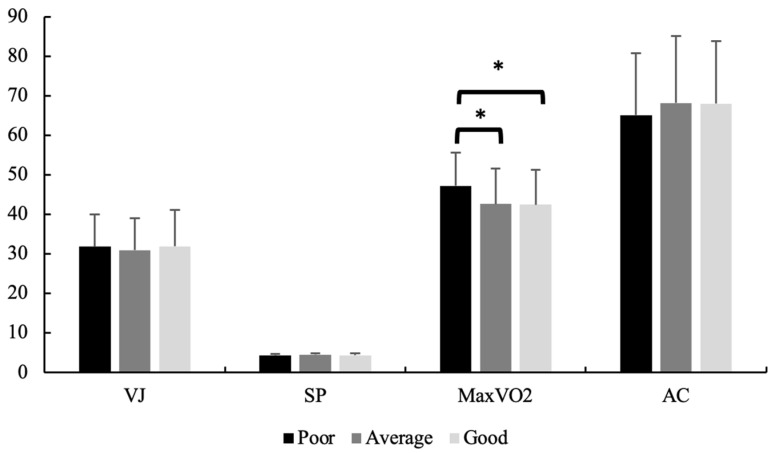
Comparison of fitness profile across different levels of academic achievement (* denotes *p* < 0.05).

We could not find a significant difference between the groups in the vertical jump, speed, and anaerobic power values based on academic achievement (F = 0.382, *p* > 0.05; F = 1.410, *p* = 0.2; and F = 1.097, *p* > 0.05, respectively). However, we found a significant difference across the groups in VO_2Max_ by academic achievement (F = 8.938, *p* < 0.001; η^2^ = 0.056). Accordingly, the poor academic achievement group had a significantly higher mean VO_2Max_ value than the average academic (47.1 vs. 42%, 6; *p* < 0.001) and good groups (47.1 vs. 42%, 4; *p* < 0.001) (Figure 4).

Table 3 shows that relationships between academic achievement and SBP, DBP, RHR, VJ, SP, VO_2Max,_ and AC did not differ by gender (*p* > 0.05). In other words, we could not conclude that gender had a significant effect on the relationship between academic achievement and the specified variables.

## 4. Discussion

Accordingly, our findings revealed that the systolic–diastolic blood pressures and heart rates of the groups were within the reference values [56]. Therefore, it was demonstrated that all three academic achievement groups have blood pressure values within the normal limits. It can be stated that only the group with lower diastolic baseline values within these three groups exhibited lower academic achievement. This situation could contribute to viewing research conducted on the subject from a different perspective. This is because research tends to focus on the relationship between high blood pressure and cognitive function. For example, previous research has suggested that higher blood pressure is associated with decreased cognitive performance among children and young adults [57,58] and cognitive decline in adults [59,60]. Similarly, the existing literature presents studies that establish a link between hypertension and decreased cognitive function [32,61,62,63]. In this study, no statistically significant differences in systolic blood pressure or heart rate were found between the groups. However, it was determined that the poor academic achievement group had significantly lower diastolic pressure compared to the average and good academic performance groups. Additional experimental research may be necessary to further investigate this topic.

Additionally, our results indicated that there were no significant variations in the vertical jump, speed, or anaerobic power values between the groups based on their academic achievement. In contrast, Zhai et al. discovered that individuals in the high academic achievement group outperformed other groups in several physical fitness assessments, including boys excelling in the 50 m sprint, standing long jump, push-ups, and 1000 m run, and girls demonstrated superior performance in the timed shuttle and 800 m jog [11]. Furthermore, Zhai et al. revealed a positive association between endurance/explosive strength and academic achievement among male and female undergraduate students [11]. Matejek and Planinšec found that children with better academic achievement performed better on most physical fitness tests than those with poor academic performance [64]. These inconsistencies across different age groups, including children, adolescents, and young adults, could potentially be attributed to age-related factors, as both muscles and the brain undergo rapid development during childhood and adolescence [65,66]. This rapid development may contribute to the difficulty of observing a discernible relationship between these variables and academic success. Hence, we posit that the observed inconsistency between our findings regarding muscular fitness and the existing literature may be attributed to the disparity in the age groups studied.

Specifically, we discovered that the poor achievement group exhibited significantly higher VO_2Max_ values than the average and good achievement groups, potentially indicating that individuals in the poor achievement group devoted more time to physical activities rather than studying. However, these results contradict the findings reported in the literature. Numerous previous studies have consistently demonstrated a correlation between aerobic fitness and academic performance [67,68,69]. In addition, a good cardiorespiratory level has been suggested to be associated with high academic achievement [70]. Longitudinal research from Germany, Sweden, Spain, Taiwan, and the United States [71,72] encompassed a cohort of 234,474 children and adolescents and evaluated their academic proficiency and aerobic capacity. The follow-up periods ranged from 1.5 to 3 years and demonstrated positive correlations between aerobic fitness and academic performance, irrespective of the duration between the initial and subsequent evaluations. No negative association was observed [73]. These findings are also consistent with the idea that cardiovascular fitness improves cognition through increased circulating factors that contribute to brain plasticity and cognitive function [16].

In this study, no significant gender differences were evident in the relationships between academic achievement and SBP, DBP, HBR, VJ, SP, VO_2Max_, and AC in our study. A few studies have shown that overall physical fitness has a positive impact on academic performance only among boys [22,69,74]. Gender differences may be influenced by several factors, such as dissimilarities in biological development and the neural pathways associated with motor and language skills in boys and girls. [11]. Another study found a significant correlation between physical fitness and reading in girls; however, this association was negative and low among boys [75]. The findings of another study suggest that the correlation between academic achievement and overall physical fitness in male and female undergraduate students is consistent. The researchers proposed that participants’ desire to achieve success in all areas might be a contributing factor [11].

The collection of data from seven secondary schools with varying socio-economic statuses and the utilization of objective measures of physiological and physical fitness can be recognized as strengths of the current study. However, there are several limitations to be considered in this research. First, given that the study had a cross-sectional design, the causation and underlying mechanism cannot be derived from the findings of this study. Second, we did not control for the effects of students’ physical activity levels at the beginning of the research. Future studies should employ a longitudinal design. Lastly, prospective researchers could consider utilizing additional instruments to collect the demographic characteristics, nutrition styles, and sleep habits of the sample.

## 5. Strengths and Limitations

The primary strength of this research lies in examining the impact of physiological factors on students’ academic achievement. This topic has been explored in only a limited number of studies. Additionally, the study specifically focused on students in the early stages of adolescence, which adds to its strength. However, this study had a few limitations. First, factors such as participants’ socio-economic status, family education level, and involvement in physical activity were not controlled for. Furthermore, this study failed to identify the cognitive functions that are believed to have a significant influence on academic achievement.

## 6. Conclusions

Our study had several major findings that emerged from our research. First, compared to the other groups based on academic achievement, the group with poor academic achievement exhibited noticeably lower diastolic blood pressure. However, additional long-term research is necessary to explore the significance of this situation regarding academic achievement and the potential application of this discovery in predicting academic achievement while taking into account student and environmental factors. Second, only the VO_2Max_ level was higher in the poor academic achievement group than in the other groups among the physical performance measurements investigated. This might mean that students who struggle academically prefer physical pursuit to study. Finally, as a result of determining how the relationship between academic achievement, physical fitness, and physiological performance elements differs in terms of gender, it was determined that there was no significant difference. It was revealed that gender did not affect the relationship between the parameters in our sample group. Overall, the findings highlight the complexity of the relationship between physiological measures, physical performance, and academic achievement, emphasizing the need for comprehensive longitudinal studies and a consideration of various factors in understanding this relationship.

## Figures and Tables

**Table 1:** Sample distribution by age and gender and descriptive data of anthropometric features.

	13 Years		14 Years		Total		Anthropometric Features	$\bar{X}$ ± SD
	*n*	%	*n*	%	*n*	%		
Boys	142	46.7	20	6.6	162	53.3	Height (cm)	158.05 ± 5.12
Girls	126	41.5	16	5.2	142	46.7	Body mass (kg)	52.95 ± 4.25
Total	268	88.2	36	11.8	304	100	BMI (kg·m^−2^)	14.98 ± 4.16

**Table 2:** Comparison of the physical fitness and cardiorespiratory profile across the academic performance levels.

Variables	Academic Achievement Levels	$\bar{X}$ ± SD	95% Confidence Interval		*p*-Value
			Lower	Upper	
SBP (mmHg)	Poor	116.1 ± 15.1	113.0	119.2	0.06
	Average	118.4 ± 17.0	115.3	121.5	
	Good	117.5 ± 14.5	114.5	120.5	
DBP (mmHg)	Poor	66.7 ± 9.36	64.8	68.6	0.04
	Average	70.9 ± 7.9	69.4	72.3	
	Good	71.5 ± 10.6	69.3	73.7	
RHR (bpm)	Poor	86.1 ± 14.5	83.2	89.1	0.48
	Average	88.2 ± 12.3	85.9	90.4	
	Good	88.3 ± 15.4	85.1	91.5	
VJ (cm)	Poor	31.8 ± 8.1	30.1	33.5	0.68
	Average	30.9 ± 8.1	29.4	32.4	
	Good	31.8 ± 9.2	29.9	33.7	
SP (s)	Poor	4.3 ± 0.4	4.2	4.4	0.24
	Average	4.4 ± 0.4	4.3	4.5	
	Good	4.4 ± 0.4	4.3	4.5	
VO_2Max_ (mL/kg/min)	Poor	47.1 ± 8.5	45.4	48.9	0.05
	Average	42.6 ± 9.0	40.9	44.2	
	Good	42.4 ± 8.9	40.5	44.3	
AC (kg m/s)	Poor	65.1 ± 15.7	61.9	68.3	0.33
	Average	68.2 ± 16.9	65.0	71.3	
	Good	67.9 ± 15.9	64.6	71.2	

Note. SD = std. deviation; SBP = systolic blood pressure; DBP = diastolic blood pressure; RHR = resting heart rate; VJ = vertical jump; SP = sprint; AC= anaerobic power.

**Table 3:** Correlations between blood pressure, RHR, physical fitness parameters, and academic achievement scores by gender.

Academic Achievement			
	Total (*n* = 304)	Boys (*n* = 162)	Girls (*n* = 142)
SBP	0.095	0.087	0.061
DBP	0.261 **	0.265 **	0.129
RHR	0.099	−0.006	0.152
VJ	−0.018	0.166 *	0.105
SP	0.078	−0.111	−0.126
VO_2Max_	−0.282 **	−0.082	−0.017
AC	0.102	0.265 **	0.061

SBP = systolic blood pressure; DBP = diastolic blood pressure; RHR = resting heartbeat rate; VJ = vertical jump; SP = sprint; AC = anaerobic power. * Correlation is significant at the 0.05 level. ** Correlation is significant at the 0.01 level.

## Data Availability

The data presented in this study are available on request from the corresponding author. The data are not publicly available due to restrictions related to ongoing work for further publications.
